# The Outcomes of Extracorporeal Shock Wave Lithotripsy for High-Density Renal Stone on Non-Contrast Computed Tomography

**DOI:** 10.7759/cureus.13271

**Published:** 2021-02-10

**Authors:** Sami Ullah, Syed Razi Muhammad, Rizwan Farooque, Umar Farooque, FNU Farukhuddin, Muhammad Daim Bin Zafar, Chinmay Khadke, Ahmad Usman, Julio Perez, Mostafa A Shehata

**Affiliations:** 1 Urology, Muhammad Medical College, Mirpur Khas, PAK; 2 Internal Medicine, Sindh Medical College, Karachi, PAK; 3 Neurology, Dow University of Health Sciences, Karachi, PAK; 4 Neurology, University Hospitals Cleveland Medical Center, Cleveland, USA; 5 Internal Medicine, Dow University of Health Sciences, Civil Hospital Karachi, Karachi, PAK; 6 Internal Medicine, Rural Medical College (Pravara Institute of Medical Sciences) Pravara Rural Hospital, Loni, IND; 7 General Surgery, Nishtar Medical University and Hospital, Multan, PAK; 8 Internal Medicine, Abrazo Community Health Network, Phoenix, USA; 9 Medicine and Surgery, Alexandria Faculty of Medicine, Alexandria, EGY

**Keywords:** outcomes, non-contrast computed tomography, high-density renal stone, extracorporeal shock waves lithotripsy, renal stone

## Abstract

Introduction

Urinary lithiasis is usually managed by extracorporeal shock wave lithotripsy (ESWL). Patients are examined using non-contrast computed tomography (NCCT) in order to evaluate the feasibility of ESWL, according to where the stone is located and how big is its size. The objective of this study is to determine the outcomes of ESWL in patients having high-density renal stone, evaluated using NCCT.

Materials and methods

A descriptive case series study was conducted in the Department of Urology, Sindh Institute of Urology & Transplantation, Karachi for six months. Patients of either gender aged between 25-50 years, who presented with solitary renal and ureteric calculi of 0.5-2 cm in diameter and high-density renal stones [>750 hounsfield units (HU)] were enrolled. ESWL was performed and a satisfactory outcome was defined as complete stone clearance in less than or equal to three ESWL sessions. Statistical Package for Social Sciences (SPSS) (IBM Corp., Armonk, NY) was used to analyze frequencies and percentages of the number of ESWL sessions, complete renal stone clearances, and satisfactory outcomes at the end of 12 weeks. A p-value of <0.05 was considered significant.

Results

The mean age of the patient was reported to be 34.08 ± 9.53 years. 51.6% male preponderance was noticed. Renal and ureteric stones were found in 69.7% and 30.3% of patients, respectively. 21.3% of patients showed stone clearance after two ESWL sessions, 27% of patients after three ESWL sessions, and 51.6% of patients after four ESWL sessions. Stone clearance was found in 58.2% of patients and a satisfactory outcome was found in 42.6% of patients.

Conclusions

Our results signify a satisfactory outcome of extracorporeal shock wave lithotripsy for high-density renal stone on non-contrast computed tomography. Further studies on a larger scale are needed to validate these results.

## Introduction

Extracorporeal shock wave lithotripsy (ESWL) continues to be one of the most accepted and used therapies for the treatment of urinary lithiasis [1]. ESWL along with percutaneous nephrolithotomy has successfully replaced open surgery procedures due to their non-invasive nature [2]. Computed tomography (CT) without contrast is the method of choice for the evaluation and selection of the patients who will eventually be subjected to this procedure. The decision is determined mainly by the size and location of the stone [3, 4]. Numerous studies have described predictive factors of pre-operative success for ESWL, mainly based on the findings of the CT without contrast [5]. The success factors have been reported as skin-to-stone distance, the body mass index (BMI), and the density of the calculus [6, 7].

Calculi are differentiated from blood clots and tumors by non-contrast computed tomography (NCCT) which measures the substance in hounsfield units (HU) [8, 9]. It provides greater density discrimination and is now the preferred method to evaluate patients with renal colic [10]. Its ability to detect density differences as low as 0.5% has been exploited to determine the composition and fragility of urinary stones [11, 12]. Clinical outcomes in ESWL are affected by the density and composition of the stone.

A study reported 41 (72%) patients with calculi of >750 HU required three or more ESWL sessions and 37 (65%) had complete clearance. Of patients with calculus diameters of >1.1 cm, 23 (77%) needed three or more ESWL sessions with a clearance rate of 60% at the end of 12 weeks [13]. However, as a result of a great heterogeneity in the definitions of success and outcome, different success rates (46-91%) have been published [5, 7, 9, 10, 14]. Therefore, this study aims to assess the outcomes of ESWL on renal stones of >750 HU assessed on NCCT and provide clarity to health care professionals with confidence.

## Materials and methods

This six-month-long descriptive case series study was conducted at Sindh Institute of Urology & Transplantation, Karachi from February 2020 to July 2020. The sample size of 122 patients was calculated using the proportion of complete stone clearance of 28%, the confidence level of 95%, and the margin of error of 5%. Patients were sampled by a non-probability consecutive sampling technique. Patients of either gender, between the age of 25 and 50 years who were presented with solitary renal and ureteric calculi of 0.5-2 cm in diameter on a plain film and high-density renal stones (>750 HU) assessed on NCCT were included in the study only if the duration of lithiasis was less than or equal to three months. Patients with ureteric and inferior calyceal calculi of >2 cm, having a solitary functioning kidney, a congenital anomaly, requiring a stent, developing steinstrasse during the therapy, suffering from a bleeding disorder, or having a BMI >30 kg/m^2^ were ejected from the study. Informed consent was taken from the patients for inclusion in the study after explaining the pros and cons of the procedure and confidentiality ensured. All patients underwent ESWL under analgesia and sedation performed by residents of the third year or above. Consecutive ESWL sessions were 14 days apart, with 3000 shock waves delivered in each session. Each ESWL session was followed by an X-ray to determine fragmentation, position, and clearance. The outcome was measured at the end of 12 weeks after the last ESWL session. A satisfactory outcome was defined as complete stone clearance in less than or equal to three ESWL sessions.

Data were analyzed using Statistical Package for Social Sciences (SPSS) version 19 (IBM Corp., Armonk, NY). Mean ± standard deviation was calculated for age, size of the stone, stone density, and duration of stone. Frequencies and percentages were calculated for gender, site of stone (renal/ureteric) complete clearance, number of ESWL sessions, and satisfactory outcome. Stratification of age, gender, duration, site, and size of the stones was done to see the effect of these on outcomes. The chi-square test was applied and a p-value of ≤0.05 was taken as significant.

## Results

The mean age of the patients was recorded to be 34.08 years with a standard deviation of 9.53 years, the minimum age recorded was 25 years and the maximum age recorded was 49 years as shown in Table 1. 57.4% (70/122) of the patients were recorded to be in less than 35 years of age group. Male preponderance was observed in the study with 51.6% (63/122) of the study population were males and 48.4% (59/122) were females.

**Table 1:** Age of the patients (n = 122) SD: Standard deviation

	Mean	SD	Minimum	Maximum
Age of the patients (years)	34.08	9.53	25	49

As shown in Table 2, the mean stone size was recorded as 1.51 cm with a standard deviation of 0.5 cm. Stone size of 51.6% of the patients (63/122) was recorded as >1 cm whereas the mean stone density was 772 HU with a standard deviation of 22.2 HU. There were 97.5% (119/122) patients with a stone density of ≤800 HU. The mean duration of stone was recorded as 2.07 months with the standard deviation of 0.31 months and 91% (111/122) of patients had a stone duration of less than or equal to two months. Renal stone was found in 69.7% (85/122) of the study population while the ureteric stone was found in 30.3% (37/122).

**Table 2:** Stone demographics (n = 122) SD: Standard deviation HU: Hounsfield units

	Mean	SD	Minimum	Maximum
Stone density (HU)	772.63	22.2	760	880
Size of the stone (cm)	1.51	0.5	1	2
Duration of stone (months)	2.07	0.31	1	3

There were 21.3% (26/122) of patients who required two ESWL sessions, 27% (33/122) of patients required three ESWL sessions, and 51.6% (63/122) of patients required four ESWL sessions. Stone clearance was found in 71 of 122 (58.2%) patients and a satisfactory outcome was found in 52 of 122 (42.6%) patients. Stratification was done to see the effect of age, gender, size of the stone, location of the stone, and duration of stone on the satisfactory outcome. Chi-square test was applied and statistically significant effect of age and size of the stone was found as p-value was found to be <0.05, as demonstrated in Table 3.

**Table 3:** Demographics of satisfactory outcomes (n = 122)

Variables		Satisfactory outcome		Total	p-value
		Yes	No		
Age group (years)	≤35	37 (52.9%)	33 (41.7%)	70 (100%)	0.006
	>35	15 (28.8%)	37 (71.2%)	52 (100%)	
	Total	52 (42.6%)	70 (57.4%)	122 (100%)	
Gender	Male	29 (46%)	34 (54%)	63 (100%)	0.273
	Female	23 (39%)	36 (61%)	59 (100%)	
	Total	52 (42.6%)	70 (57.4%)	122 (100%)	
Duration of stone (months)	≤2	48 (43.2%)	63 (56.8%)	111 (100%)	0.757
	>2	4 (36.4%)	7 (63.6%)	11 (100%)	
	Total	52 (42.6%)	70 (57.4%)	122 (100%)	
Stone size (cm)	≤1	12 (20.3%)	47 (79.7%)	59 (100%)	0.001
	>1	40 (63.5%)	23 (36.5%)	63 (100%)	
	Total	52 (42.6%)	70 (57.4%)	122 (100%)	
Location of stone	Renal	40 (47.1%)	45 (52.9%)	85 (100%)	0.165
	Ureteric	12 (32.4%)	25 (67.6%)	37 (100%)	
	Total	52 (42.6%)	70 (57.4%)	122 (100%)	

## Discussion

Skin-to-stone distance greater than 10 cm and BMI reduces the effectiveness of lithotripsy [5]. Evidence suggests that some lithotripters are less effective than others as treatment protocol and experience of the operator can affect the outcomes [15]. In this study, however, a satisfactory outcome was found in 42.6% of patients while stone clearance was found in 58.2% of patients. A study reported that patients treated by ESWL had an increased incidence of diabetes mellitus and were more likely to develop new-onset hypertension [16]. Patients older than 60 years were found to have an elevated intrarenal resistive index in the study by Janetschek et al. [17]. These findings imply that ESWL treatment can have critical and persistent side effects in the elderly.

Another study reported a high risk of patients developing hypertension (p-value = 0.034) and diabetes mellitus (p-value < 0.001) after ESWL treatment in comparison to the control group. The study also reported a significant correlation of developing hypertension with bilateral ESWL treatments (p-value = 0.033). Multivariate analysis in the same study showed risk associated with obesity (p-value = 0.003) and an increase in BMI in patients (p-value = 0.003). The number of shocks administered was found to be significantly related to an increased risk of developing diabetes mellitus in patients with a history of ESWL (p-value = 0.005). Acute symptomatic pancreatitis after ESWL has also been reported. Therefore, ESWL can affect the pancreas as well [18]. Increased serum lipase, amylase, and urinary amylase in the first seven days following ESWL of proximal renal and ureteral stones have been recorded, which were not increased in lower ureteral stone treatment [19].

Some studies have predicted factors that affect ESWL’s success in patients to be governed by the findings of the NCCT [5]. Success factors have been reported to be the stone-to-skin distance, the BMI, and the calculus density [6, 7]. The composition and density of the stone vary which affects the fragility of a calculus, inevitably affecting clinical outcome in ESWL.

There were 21.3% patients with two ESWL sessions, 27% patients with three ESWL sessions, and 51.6% patients with four ESWL sessions in this study. However, in a different study of patients with calculi of >750 HU, 72% required three or more ESWL sessions and 65% had complete clearance. In patients with calculus diameters of >1.1 cm, 77% needed three or more ESWL sessions and the clearance rate was 60% at the end of 12 weeks [13].

## Conclusions

Extracorporeal shock waves lithotripsy for high-density renal stone on non-contrast computed tomography was found to be satisfactory. Whereas, increasing age and stone size had a significant correlation with a satisfactory outcome. We suggest replicating these findings on a larger sample population to yield more specific results.
